# Comparing the Efficacy of Combined Treatment with Medial Branch Block and Facet Joint Injection in Axial Low Back Pain

**DOI:** 10.1155/2021/1343891

**Published:** 2021-01-07

**Authors:** Jeong-Hwan Seo, Sung-Woon Baik, Myoung-Hwan Ko, Yu-Hui Won, Sung-Hee Park, Sang Wook Oh, Gi-Wook Kim

**Affiliations:** ^1^Department of Physical Medicine & Rehabilitation, Jeonbuk National University Medical School, Jeonju 54907, Republic of Korea; ^2^Research Institute of Clinical Medicine of Jeonbuk National University-Biomedical Research Institute of Jeonbuk National University Hospital, Jeonju 54907, Republic of Korea; ^3^Institute of Science Education, Division of Science Education, Jeonbuk National University, Jeonju 54896, Republic of Korea

## Abstract

**Introduction:**

Medial branch nerve block (MBB) and facet joint injections (FJIs) can be used to manage axial low back pain. Although there have been studies comparing the MBB and FJI effects, a few studies have compared the therapeutic effects of both interventions combined with each separate intervention. This study aimed to compare the pain relief effect of MBB, FJI, and combined treatment with MBB and FJI in patients with axial low back pain.

**Methods:**

We conducted a retrospective review of patients with axial low back pain who had chart records of the Numeric Rating Scale (NRS) and Oswestry Disability Index (ODI) scores measured before treatment and within 6 weeks after treatment. The proportion of patients with successful responses (>30%) was calculated and is presented with Wald confidence intervals.

**Results:**

We included 66 patients (33, 17, and 16 patients in the MBB, FJI, and combined treatment with MBB and FJI groups). All the patient groups showed significant posttreatment improvements in the NRS [(proportion >30% decrease: MBB 24.2% (9.6–38.9), FJI 29.4% (7.8–51.1), and MBB + FJI 25.0% (3.8–46.2)] scores and the ODI [proportion >30% decrease: MBB 39.4% (22.7–56.1), FJI 23.5% (3.4–43.7), and MBB + FJI 37.5% (13.8–61.2)] scores. Furthermore, there was no significant among-group difference in the ODI and NRS scores.

**Conclusion:**

MBB, FJI, and combined treatment with MBB and FJI can reduce axial low back pain and improve secondary functional degradation. Although combined treatment with MBB and FJI required a longer intervention time, it did not have a pain relief effect superior to that of MBB or FJI alone.

## 1. Introduction

The prevalence of chronic spinal pain with various structural etiologies in the general population is around 66%; among them, 44%, 56%, and 15% of the patients present with cervical, lumbar, and thoracic pain, respectively [1, 2]. Chronic low back pain (LBP) has a prevalence rate of around 80% in the total population; moreover, it can impair daily living in severe cases [3]. Depending on its presentation, pain can be categorized as somatic (facet joint pain, myofascial pain, and discogenic pain) or radicular (disc herniation, annular tear, and spinal stenosis).

Lumbar facet joint pain is caused by acute or chronic inflammation of a lumbar zygapophyseal joint and affects 15–45% of patients with chronic LBP [4]. Regarding general treatment, an appropriate bedrest period could be beneficial. Furthermore, physical modalities (ultrasound, shortwave diathermy, superficial moist heat and ice massage, and hydrotherapy) could help reduce surrounding muscle spasm and alleviate pain. Regarding medication, analgesics, nonsteroidal anti-inflammatory agents, muscle relaxants, and antidepressants can be used.

Furthermore, facet joint pain management can be achieved using facet joint procedures using therapeutic interventions, including fluoroscopically guided facet joint injection (FJI), medial branch block (MBB), or radiofrequency ablation [4–6]. Facet joint degeneration can cause problems, including arthritis and abnormal motion associated with disc degeneration. Moreover, the spinal facet joint has an abundant nerve supply; therefore, pain can be caused by arthritis change, degenerative change, inflammation, and injury [4, 7, 8]. FJI involves a direct injection into the facet joint capsule, which can cause problems such as inflammatory change. Moreover, pain alleviation involves an anti-inflammatory effect or direct injection of the facet joint capsule, which includes the nerve endings, causing problems. MBB achieves pain alleviation by anesthetizing the medial branches of the posterior primary rami, which are the main nerves responsible for dual innervation to the joint, and, therefore, block axonal transport or suppress nociceptive discharge [4, 9–11]. For managing axial LBP in facet joint pathology, which intervention is superior remains controversial, as both interventions are effective in managing axial LBP [6, 9, 12–15].

There have been numerous studies examining and comparing the individual effects of MBB and FJI on pain alleviation. However, only few studies have compared the effects of combined treatment with MBB and FJI with those of the individual interventions. We hypothesized that a patient's pain relief would be greater when the anti-inflammation or direct anesthesia of the facet joint capsule induced by FJI and the anesthetizing of the medial branches of the posterior primary rami caused by MBB occurred simultaneously. This study, therefore, aimed to compare the effects of FJI, MBB, and combined treatment with MBB and FJI in patients with axial LBP.

## 2. Materials and Methods

### 2.1. Study Design

In this retrospective study, we reviewed the records of patients who received FJI, MBB, or combined treatment with MBB and FJI for axial LBP between December 2016 and April 2020. This study was approved by the Institutional Review Board of our university hospital (CUH 2020-05-011).

### 2.2. Participants

The inclusion criteria were patients with axial LBP who had chart records for the Numeric Rating Scale (NRS) and Oswestry Disability Index (ODI) scores before treatment and within 6 weeks after treatment.

The exclusion criteria were patients who had NRS and ODI records obtained >6 weeks after treatment or those with missing records for pre- or posttreatment NRS or ODI scores.

### 2.3. Interventions

All interventions were performed under the control of a C-arm image intensifier (BV Pulsera mobile C-arm, Philips Healthcare Co., Ltd., the Netherlands) with the patient unsedated and in a prone position. The procedures were performed via an aseptic technique using a 6.0 cm-long 24G spinal needle.

#### 2.3.1. MBB Procedure

**Figure 1:**
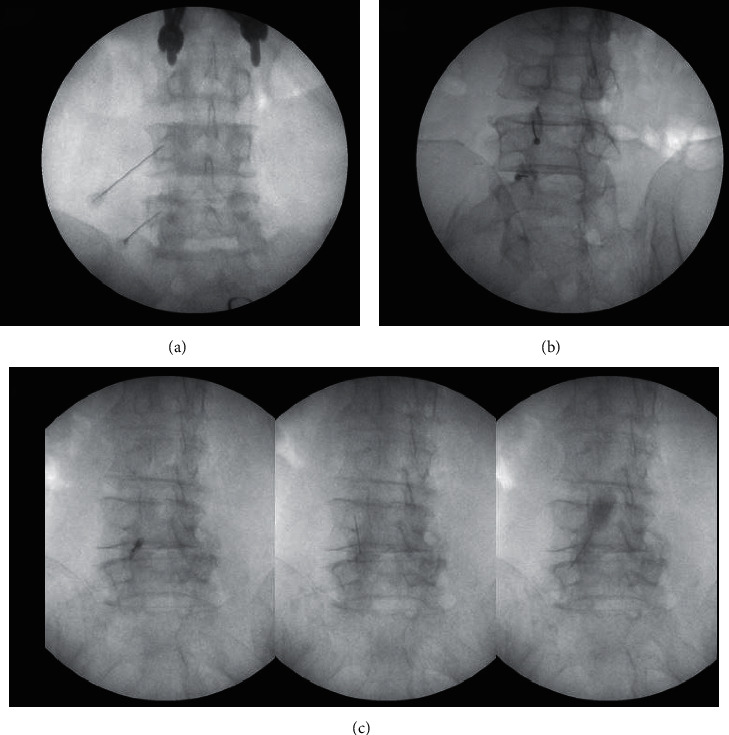
C-arm image intensifier view of MBB, FJI, and combined treatment with MBB and FJI: (a) MBB for nerves innervated into the left L4-5 facet joint, (b) FJI for the left L3-4 and L4-5 facet joints, and (c) combined treatment with MBB and FJI for the left L4-5 facet joint. MBB, medial branch block; FJI, facet joint injection.

Initially, we identified the target level in the C-arm AP view and subsequently aligned the superior and inferior endplates of the target vertebral body. Next, the C-arm was moved into an oblique view on the same side as the treatment site while ensuring visibility of the Scotty dog sign and the junction between the transverse process (TP) and superior articular process (SAP) [9, 10]. At L1–L4, the injection targets in the medial branches are located at the junctions between the SAP and TP. Here, the target nerves cross between the superior TP border and the mamilloaccessory notch, with this location being considered the eye of the Scotty dog [10]. At the L5 level, the injection target is the dorsal branch of L5, which runs superior to the ala of sacrum in a direction similar to that of the medial branches at L1–L4 [10]. Given that the injection target is located at the pedicle center, it was set slightly inferior to the ala of sacrum. Both nerve branches innervating the target joint were blocked as previously described where 1.5 cc of injectant, which contained a mixture of 2.5 mg dexamethasone and 1 cc of 1% lidocaine, was injected into each nerve (Figure 1(a)) [11, 14, 15].

#### 2.3.2. Facet Joint Injection Procedure

First, we identified the target level in the C-arm AP view. Subsequently, the image intensifier was tilt-angled towards the same side as the injection site until the silhouette of the target facet joint space could be observed and the entry site to the joint was maximally visible; the angle here was generally 5–10° [9, 10]. Radiographic contrast medium was injected into the target joint. Furthermore, after confirming proper needle placement in the target site, 1 cc of injectant, which contained 2.5 mg of dexamethasone and 0.5 cc of 1% lidocaine, was injected into each joint (Figure 1(b)) [11, 14, 16–19].

#### 2.3.3. Procedure for Combined Treatment with MBB and FJI

MBB and FJI were performed together at the injection target area using the aforementioned procedures. Dexamethasone 5 mg was mixed with 1% lidocaine where the lidocaine volume was adjusted depending on the injection site. We injected 1.5 cc and 1 cc of injectant into each medial branch innervating the target joint and each target facet joint, respectively (Figure 1(c)).

### 2.4. Outcome Measures

The primary outcome to measure the effectiveness of the intervention was the NRS score of chronic LBP. For the NRS assessment, the patients chose a number between 0 and 10 to represent their pain extent. A score of 0 indicates no pain whatsoever, while a score of 10 indicates the most severe pain imaginable [20]. For the secondary outcome, we measured the Korean version of the ODI. The ODI is a self-report questionnaire for assessing the restriction extent in daily living due to lower back or leg problems. The questionnaire consists of 10 items (pain severity, personal care, lifting objects, walking, sitting, standing, sleeping, sexual activity, social life, and travel). The total score ranges from 0 to 50 points with a higher score indicating more severe pain and restrictions in daily living [21].

### 2.5. Statistical Analysis

All statistical analyses were performed using IBM SPSS statistics version 24.0. Regarding demographic characteristics, continuous variables are presented as the mean and standard deviation and underwent between-group comparisons. Categorical variables are presented as frequencies and percentages and analyzed using a Chi-square test. For within-group comparisons, a normality test was initially performed. Subsequently, a parametric paired t-test was used for normally distributed variables, while a nonparametric Wilcoxon signed-rank test was used for nonnormally distributed variables. Between-group comparisons were performed using parametric one-way analysis of variance and a nonparametric Kruskal–Wallis test for normally and nonnormally distributed variables, respectively. For the normality test, the Shapiro–Wilk statistic was used. The proportion of patients with successful responses (>30%) was measured and is presented with Wald confidence intervals. Statistical significance was set at *p* < 0.05.

## 3. Results

### 3.1. Demographic Characteristics

There were 33, 17, and 16 patients in the MBB, FJI, and combined treatment with MBB and FJI groups, respectively.  Table 1 shows the demographic characteristics where there were no significant among-group differences in the generation ratio, age, pre-ODI, pre-NRS, and postintervention evaluation interval.

### 3.2. Changes in the NRS Score

All three groups showed a significant postintervention decrease in the NRS score (Table 2). The postintervention decrease in the mean NRS score was 1.27 ± 1.64, 1.29 ± 1.57, and 0.94 ± 1.39 in the MBB, FJI, and combined treatment with MBB and FJI groups, respectively. The proportion of patients whose NRS decreased by more than 30% compared to baseline was 39.4% (22.7–56.1), 23.5% (2.4–43.7), and 37.5% (37.5, 13.8–61.2) in the MBB group, FJI group, and MBB + FJI group, respectively. However, there were no significant among-group differences in the NRS score.

### 3.3. Changes in the ODI

Similar to the NRS score, all three groups showed a significant postintervention decrease in the ODI (Table 3). The mean decrease in the ODI from pre- to postintervention was 3.82 ± 6.62, 3.94 ± 5.97, and 5.06 ± 3.59 in the MBB, FJI, and combined treatment with MBB and FJI groups, respectively. The proportion of patients whose NRS decreased by more than 30% compared to baseline was 24.2% (9.6–38.9), 29.4% (7.8–51.1), and 25.0% (3.8–46.2) in the MBB group, FJI group, and MBB + FJI group, respectively. However, there were no significant among-group differences in the changes in the ODI.

### 3.4. Adverse Effects

In the MBB group, one patient complained of mild dizziness. One patient each in the MBB and FJI groups complained of paresthesia in the calf area and were followed up as outpatients; however, they improved without any particular treatment. None of the other patients showed any major adverse effects.

## 4. Discussion

In this study, all patients with axial LBP who underwent MBB, FJI, or combined treatment with MBB and FJI showed significant posttreatment improvements in pain scores and pain-caused daily living restrictions, as measured using the ODI. However, there were no significant among-group differences.

Fluoroscopy-guided FJI and MBB are useful diagnostic and therapeutic methods for LBP caused by facet joint pathology [13]. In FJI, although the corticosteroid effects remain unclear, they can alleviate in patients with synovitis or osteoarthritis-related inflammation via their anti-inflammatory effect [18]. In addition, post-FJI pain relief is more effective among patients presenting an inflammatory process in the facet joint on SPECT imaging [22, 23]. MBB-induced pain relief is known to be mediated by various mechanisms, including inhibiting nociceptive discharge, blocking the sympathetic reflex arc, axonal transport, sensitization, or anti-inflammatory effects [8, 24–30].

Although numerous studies have assessed the individual effects of MBB and FJI for axial chronic LBP and compared their effects, there have been very few studies comparing the MBB and FJI effects and the effects of combined treatment with MBB and FJI. This study aimed to compare the effects of the interventions individually and both combined in patients with axial LBP.

We divided the patients into three groups according to the received intervention methods and analyzed the pre- and posttreatment ODI and NRS scores. There has been previous moderate evidence that MBB and FJI provide short- and long-term pain relief for facet joint pain, which is the major cause of axial LBP [6, 31]. Similarly, we observed a significant postintervention improvement in pain in all three groups (MBB, FJI, and combined treatment with MBB and FJI). Meanwhile, a decrease of 30% or more in the NRS scores is considered moderately important, according to the initiative on methods, measurement, and pain assessment in clinical trials [32]. Although all groups showed postintervention improvement in symptoms, the proportion of patients with a decrease in NRS score by more than 30% ranged from 24 to 29% in all groups. All three groups had baseline NRS and ODI scores of 5–6 out of 10 and 21–25 out of 50, respectively, which indicate moderate pain severity and restriction of daily activities. Moreover, the intervention effect could have been lower since the intervention was only performed once. The relationship between MBB frequency and its therapeutic effects remains unclear; however, previous studies on interventions for axial LBP that measured the pain relief effect after 1–2 years with at least 1 and as many as 10 treatment rounds reported that MBB had a significant pain relief effect [11, 14, 33]. Therefore, we could have observed different results if more treatment rounds had been administered depending on the patient's symptoms.

We hypothesized that the patient's pain relief would be greater when the anti-inflammation or direct anesthesia of the facet joint capsule induced by FJI and the anesthetizing of the medial branches of the posterior primary rami caused by MBB occurred simultaneously. However, we found no significant differences in the therapeutic effects of MBB, FJI, and combined treatment with MBB and FJI. In addition, while combining the interventions that resulted in a longer treatment period, it did not lead to a superior pain-reducing effect than individual treatment.

After MBB, one patient complained of a mild headache and dizziness, which improved without any particular treatment. These symptoms could have been caused by lidocaine used for local anesthesia. Although we used only a small quantity of 1% lidocaine, its entry into a blood vessel adjacent to the target area, including the radicular artery or radicular vein, increases its blood levels. This can result in CNS-related adverse reactions, including drowsiness or psychosis, as well as vasoconstrictor reactions, including elevated heart rate and blood pressure [34, 35]. Furthermore, one patient in both the MBB and the FJI group complained of postintervention paresthesia in the calf area. Although uncommon, there have been reports of similar adverse effects from inaccurate delivery of the injectant (lidocaine and corticosteroid) to the target area or when it leaks out of the facet joint. This results in its spread to soft tissue or the intervertebral foramen in the epidural space and affects the branches of adjacent spinal nerves rather than the target nerve [36].

This study has some limitations. First, since this was a retrospective study, we could not consistently control the time between the intervention and follow-up tests to examine the treatment effects. Nevertheless, there were no significant among-group differences in the age, pre-ODI, pre-NRS, and postintervention evaluation interval (Table 1). Second, we could not perform immediate and long-term follow-ups of the effects. To more precisely understand the treatment effects, their persistence over time needs to be evaluated. Additional randomized controlled trials will be needed to accurately investigate the effects of each intervention. Finally, MBB is widely used as a diagnostic and prognostic procedure for radiofrequency ablation and is mainly used when radiofrequency ablation is not feasible.

## 5. Conclusions

MBB, FJI, and combined treatment with MBB and FJI are all effective at reducing axial LBP, as well as at improving pain-induced secondary functional degradation. Although combined treatment with MBB and FJI requires a longer intervention time, it did not show an effect superior to that of MBB or FJI.

## Figures and Tables

**Table 1:** Preintervention demographic characteristics of patients.

	MBB (*N* = 33)	FJI (*N* = 17)	MBB + FJI (*N* = 16)	Total (*N* = 66)	*p* value
Sex (male: female)	15 (45.5%): 18 (54.5%)	9 (52.9%): 8 (47.1%)	6 (37.5%): 10 (62.5%)	35 (46.7%): 40 (53.3%)	0.673
Age	61.09 ± 13.47	63.65 ± 11.19	59.50 ± 16.43	60.89 ± 13.27	0.679
ODI	24.45 ± 7.10	24.12 ± 6.63	21.44 ± 9.49	23.27 ± 7.62	0.417
NRS score	5.85 ± 1.54	6.06 ± 1.68	5.13 ± 2.09	5.72 ± 1.67	0.346
Evaluation interval	18.00 ± 10.02	16.29 ± 10.70	12.81 ± 9.03	22.89 ± 27.37	0.241

(1) Chi-square test, (2) one-way ANOVA, 3) Kruskal–Wallis test. MBB, medial branch block; FJI, facet joint injection; MBB + FJI, combined treatment with MBB and FJI; ODI, Oswestry Disability Index; NRS, Numeric Rating Scale.

**Table 2:** Changes in the NRS score in the three groups before and after the intervention.

NRS	Before intervention	After intervention	Δ	Proportion of patients with >30% decrease in the NRS score (%, CI)	Intra-*p* value	Inter-*p* value
MBB (*N* = 33)	5.85 ± 1.54	4.58 ± 1.90	1.27 ± 1.64	39.4, 22.7–56.1	<0.001^†*∗*^	0.774^‡^
FJI (*N* = 17)	6.06 ± 1.68	4.76 ± 1.75	1.29 ± 1.57	23.5, 3.4–43.7	0.003^†*∗*^	
MBB + FJI (*N* = 16)	5.13 ± 2.09	4.19 ± 2.07	0.94 ± 1.39	37.5, 13.8–61.2	0.028^†*∗*^	

^†^Wilcoxon signed-rank test of difference. ^‡^Kruskal–Wallis test. ΔPrepost. *∗* Statistical significance. MBB, medial branch block; FJI, facet joint injection; MBB + FJI, combined treatment with MBB and FJI; NRS, Numeric Rating Scale; CI, confidence interval.

**Table 3:** Pre- and postintervention changes in the ODI in the three groups.

ODI	Before intervention	After intervention	Δ	Proportion of patients with >30% decrease in the ODI (%, CI)	Intra-*p* value	Inter-*p* value
MBB (*N* = 33)	24.45 ± 7.10	20.64 ± 8.24	3.82 ± 6.62	24.2, 9.6–38.9	0.002^†*∗*^	0.774^‡^
FJI (*N* = 17)	24.12 ± 6.63	20.18 ± 10.00	3.94 ± 5.97	29.4, 7.8–51.1	0.015^†*∗*^	
MBB + FJI (*N* = 16)	21.44 ± 9.49	16.38 ± 7.47	5.06 ± 3.59	25.0, 3.8–46.2	<0.001^†*∗*^	

^†^Paired t-test. ^‡^One-way ANOVA. ΔPrepost. *∗*Statistical significance. MBB, medial branch block; FJI, facet joint injection; MBB + FJI, combined treatment with MBB and FJI; ODI, Oswestry Disability Index; CI, confidence intervals.

## Data Availability

The data used to support the findings of this study are available from the corresponding author upon request.
